# Supplementary material for: Legislation on Medical Assistance in Dying (MAID): Preliminary Consideration on the First Regional Law in Italy
Source: Healthcare (Basel). 2025 May 7;13(9):1091. doi: 10.3390/healthcare13091091 (PMC12071898; doi:10.3390/healthcare13091091)
Supplement: Supplementary file 1 [file healthcare-13-01091-s001.zip › healthcare-3604279-supplementary.pdf]

Tuscany Region

REGIONAL LAW MARCH 14, 2025, NO. 16

Organizational modalities for the implementation of Constitutional Court rulings 242/2019 and 135/2024.

TABLE OF CONTENTS

PREAMBLE

Art. 1 - Purpose.

Art. 2 - Requirements for access to medically assisted suicide.

Art. 3 - Establishment of the Permanent Multidisciplinary Commission.

Art. 4 – Modalities of access to medically assisted suicide.

Art. 5 - Verification of requirements

Art. 6 - Methods of implementation.

Article 7 - Support for the implementation of the medically assisted suicide procedure

Article 8 - Gratuity of benefits

Art. 9 - Financial regulation

PREAMBLE

(Paraphrased by the authors) It places references to the constitutional provisions regulating the division of legislative competence between the State and the Regions and the right to health, rules on the organization of the national (Legislative Decree No. 502 of December 30, 1992) and regional health service in Tuscany (Regional Law No. 40 of February 24, 2005), provisions to ensure access to palliative care and pain therapy (Italian Law n. 38/2010) and rules on informed consent and advance treatment arrangements (Italian Law 219/2017).

… and considering that:

1. Italian Region of Tuscany, including in the implementation of this law, shall protect the dignity of life of the person in compliance with the Constitution of the Italian Republic and in accordance with the laws of the State, guaranteeing, even in the terminal phase of life, the necessary health care in compliance with law n. 38/2010, as well as, within public facilities, psychological support and, when required, spiritual or lay assistance;

2. the Constitutional Court intervened in its immediately executable Judgment 242/2019, which identified a circumscribed area in which the incrimination of aiding someone in suicide - under Article 580 of the Criminal Code - does not comply with the Constitution, corresponding specifically to cases where the would-be suicide is identified as a person "(a) suffering from an irreversible pathology and (b) a source of physical or psychological suffering, which he or she finds absolutely intolerable, who is (c) kept alive by means of life-support treatment, but remains (d) capable of making free and conscious decisions",

3. Moreover, in its ruling 135/2024, the same Constitutional Court pointed out that there can be no "distinction between the situation of a patient who is already on life-support treatment, whose termination he or she may demand, and that of a patient who, in order to survive, needs, based on medical assessment, the activation of such treatment, which, however, he or she may

refuse",

4. the Constitutional Court expressly refers to law 219/2017 which provides that the patient can already decide to let himself die by requesting the discontinuation of life-support treatments and undergoing continuous deep sedation, which places him/her in a state of unconsciousness until death. Decision that the physician is obliged to respect;

5. Furthermore, in the context of Judgment 242/2019, the Constitutional judges held that the verification of the conditions that make aid to suicide legitimate and the modalities of its execution should remain entrusted, pending legislative intervention, to public facilities of the national health service, and that for this purpose the opinion of the territorially competent ethics committee should be acquired. This is in line with what has already been established in previous pronouncements regarding similar situations;

6. With this law, the Region, in the exercise of its powers in the field of health protection, and in implementation of an immediately enforceable ruling, dictates rules of an organizational and procedural nature to uniformly regulate in its territory the exercise of the functions that constitutional jurisprudence attributes to health care facilities;

7. the introduction of the present law serves to define the times and modalities regarding the procedure indicated by the Constitutional Court and, therefore, to eliminate any uncertainty and problems with respect to the provision of a health care service divided into several stages, from the verification of the access conditions to the verification of the modalities of self-administration of the drug that can guarantee a rapid, painless and dignified death. Timing and procedures are, in fact, key elements for the faculty recognized by the Constitutional Court to be effectively usable, accessing conditions of illness, suffering and extreme urgency;

8. this law in any case recognizes its own supersession with respect to a subsequent State legislation regulating the matter, establishing its fundamental principles;

Approves this law

Art. 1

Purpose

1. The Italian Region of Tuscany, in the exercise of its competencies, regulates the organizational modalities for the implementation of the provisions of the Constitutional Court's rulings September 25, 2019, No. 242 and July 1, 2024, No. 135, regarding medically assisted suicide.

Art. 2

Requirements access to medically assisted suicide

1. Until the state regulations come into force, individuals who meet the requirements indicated by Constitutional Court rulings 242/2019 and 135/2024 (*See point 2 in the Preamble*) can access the procedures related to medically assisted suicide, in the modalities stipulated in Articles 1 and 2 of Law No. 219 of December 22, 2017 (Rules on informed consent and advance treatment arrangements).

Art. 3

Establishment of the permanent multidisciplinary commission

1. Within 15 days of the enactment of this law, local health units shall establish a permanent multidisciplinary commission (hereinafter referred to as the Commission) to verify the existence of the requirements for access to medically assisted suicide as well as to verify or define the modalities for its implementation.

2. The Commission consists of the following members:

a) A palliative care physician with direct care skills and experience;
b) A psychiatric physician;
c) An anesthesiologist physician .
d) A psychologist;
e) A legal doctor;
f) A nurse.

3. The commission is supplemented from time to time by a physician who

is a specialist in the disease from which the person requesting access to medically assisted suicide is affected.

4. The members are identified, on a voluntary basis, from among the staff employed by the local health unit. In case of unavailability of internal staff, members may be identified from employees of other facilities or entities of the regional health service.

5. Participation in the Commission does not entail the payment of any fee or attendance allowance. This is without prejudice to the reimbursement of expenses incurred, within the limits laid down for the employee, by the local health unit where the Commission is established. Participation in the Commission is considered an institutional activity to be carried out during working hours.

Art. 4

Modalities of access to the medically assisted suicide

1. The person concerned, or his/her representative, submits an application to the local health unit with territorial jurisdiction to ascertain the requirements for access to medically assisted suicide as well as for approval or definition of the relevant implementation arrangements.

2. The application shall be accompanied by the available health records. If necessary, the application may be accompanied by the indication of a trusted physician and the protocol referred to in Article 6, paragraph 2.

3. The local health unit shall promptly transmit the application and related documentation to the Commission and the Committee of Clinical Ethics (hereinafter referred to as the Committee) operating at the facility in accordance with Article 99 of Regional Law No. 40 of February 24, 2005 (Rules of the Regional Health Service).

Art. 5 Verification of requirements

6. The procedure for verifying the requirements set forth in Article 2, Paragraph 1, shall be concluded within 20 days after receiving the application

by means of the notice referred to in Paragraph 6. The deadline may be suspended once, for a period not exceeding 5 days, for clinical-diagnostic investigations.

1. The Commission preliminarily verifies that the applicant has received clear and adequate information about the possibility of accessing a palliative care pathway. The applicant is also informed of his or her right to refuse or withdraw consent to any health treatment, including life support, and of the possibility of access deep palliative sedation according to law 219/2017.

2. If the applicant confirms the desire to access medically assisted suicide, the Commission proceeds to verify the requirements. To this end, the Commission examines the alleged documentation and carries out the assessments that are necessary, also with the support of the facilities of the regional health service, ensuring personal and direct interlocution with the person concerned, also in consultation with a physician that may be indicated by the person. Consent to medically assisted suicide must be free and informed in accordance with Article 1, Paragraph 4 of Law 219/2017.

3. The Commission shall request the Committee's opinion on the ethical aspects of the case under consideration by forwarding to it the documentation pertaining to the interlocution and the findings. The Committee shall render its opinion within seven days of receipt of the documentation.

4. The Commission must request the Committee's opinion in time to ensure that compliance with the deadline in paragraph 4 is compatible with the overall deadline for the procedure conclusion referred to in paragraph 1.

5. The Commission prepares the final report stating the results of the assessment of the requirements. The local health unit shall notify the person concerned of the results of the assessment.

Art. 6 Methods of implementation

1. In case of a positive verification of the requirements, the Commission shall proceed in accordance with paragraphs 2 and 3 for the purpose of approving or defining the modalities for the implementation of medically assisted suicide. The procedure shall be concluded within ten days after the

communication referred to in Article 5, Paragraph 6, with the communication of the results provided for in Paragraph 7.

2. The person concerned may apply to the Commission for approval of a protocol drawn up by the trusted physician containing the procedures for implementing medically assisted suicide.

3. The person concerned may also request the Commission to define, in collaboration with them, the modalities for implementing medically assisted suicide by drafting an appropriate protocol. If an agreement cannot be reached, the request will not be processed.

4. Implementation arrangements must provide for physician assistance and be such as to avoid abuse to vulnerable people, to ensure the dignity of the patient, and to prevent the patient from suffering.

5. The Commission shall seek the opinion of the Committee on the adequacy of the protocol referred to in paragraphs 2 and 3. The Committee shall give its opinion within 5 days of receipt of the documentation submitted by the Commission.

6. The Commission must request the Committee's opinion in time to ensure that compliance with the time limit in paragraph 5 is compatible with compliance with the overall deadline for the conclusion of the procedure referred to in paragraph 1.

7. The Commission shall prepare the final report on the outcomes of the application referred to in paragraphs 2 and 3. The local health unit shall notify the applicant of the outcomes of the procedure.

Art. 7

Supporting the implementation of the medically assisted suicide procedure

1. Within 7 days of the communication referred to in Article 6, paragraph 7, the local health unit shall ensure, in the modalities provided for in the protocol approved by the Commission or defined by it in agreement with the person concerned, technical and pharmacological support as well as health care assistance for self-administration of the authorized drug. The assistance is

provided by health personnel on a voluntary basis and is considered an institutional activity to be carried out during working hours.

2. The practices and treatments regulated by this law constitute a level of health care superior to the essential levels of care. The Region shall meet with its own resources the financial effects related to these benefits and treatments, in accordance with the provisions of Article 13 of Legislative Decree No. 502 of December 30, 1992 (Reorganization of the discipline in health care, pursuant to Article 1 of Law No. 421 of October 23, 1992).

3. The eligible person authorized to access medically assisted suicide can decide at any time to suspend or cancel the provision of treatment.

4. In any case, local health units shall conform the procedures governed by this law to state regulations.

Art. 8 Gratuity of benefits

1. The practices and treatments provided by the regional health service as part of the medically assisted suicide care pathway are free of charge.

Art. 9 Financial rule

1. For the implementation of the practices and treatments provided by this law as part of the therapeutic and assistance pathway of medically assisted suicide, an expenditure of 10,000.00 euros is estimated for each of the years 2025, 2026 and 2027, which is to be met from the appropriations of the Mission 12 "Social Rights, Social Policies and Family," Program 02 "Disability Interventions," Title 1 "Current Expenditures," of the 2025 - 2027 budget, annualities 2025, 2026 and 2027.

2. Charges for subsequent years are met by budget law
